# Photodepletion with 2-Se-Cl prevents lethal graft-versus-host disease while preserving antitumor immunity

**DOI:** 10.1371/journal.pone.0234778

**Published:** 2020-06-22

**Authors:** Jason M. Grayson, Mildred D. Perez, Rebecca Blevins, Benjamin N. Coe, Michael R. Detty, Zachariah A. McIver

**Affiliations:** 1 Department of Microbiology and Immunology, Wake Forest University School of Medicine, Winston-Salem, North Carolina, United States of America; 2 Department of Hematology and Oncology, Wake Forest University School of Medicine, Winston-Salem, North Carolina, United States of America; 3 Department of Chemistry, University at Buffalo, The State University of New York, Buffalo, New York, United States of America; University of Iowa, UNITED STATES

## Abstract

Acute graft-versus-host-disease (GVHD), limits the use of hematopoietic cell transplant (HCT) to treat a variety of malignancies. Any new therapeutic approach must satisfy three requirements: 1) Prevent GVHD, 2) Maintain anti-pathogen immunity, and 3) Maintain anti-tumor immunity. In prior studies we have shown that the selective photosensitizer 2-Se-Cl eliminates highly alloreactive lymphocytes from the graft prior to HCT preventing GVHD and that antiviral immune responses were preserved following incubation with 2-Se-Cl. In this report, we investigated whether 2-Se-Cl treatment preserves antitumor immunity, and then used high dimensional flow cytometry to identify the determinants of successful immune reconstitution. Donor C57BL/6 splenocytes were cocultured for 4 days with irradiated BALB/c splenocytes and then exposed to 2-Se-Cl. Photodepletion (PD)-treated splenocytes were then infused into lethally irradiated BALB/c mice inoculated with A20 leukemia/lymphoma cells. Recipient mice that received PD-treated splenocytes survived > 100 days without evidence of GVHD or leukemia. In contrast, mice that did not receive PD-treated cells at time of HCT died of leukemia progression. Multiparameter flow cytometry of cytokines and surface markers on peripheral blood samples 15 days after HCT demonstrated unique patterns of immune reconstitution. We found that before clinical disease onset GVHD was marked by functionally exhausted T cells, while tumor clearance and long-term survival were associated with an expansion of polyfunctional T cells, monocytes, and DCs early after transplantation. Taken together these results demonstrate that 2-Se-Cl photodepletion is a new treatment that can facilitate HCT by preventing GVHD while preserving antiviral and anti-tumor immunity.

## Introduction

Acute graft-versus-host disease (GVHD) is a significant barrier to successful hematopoietic cell transplantation (HCT), and is associated with delayed immune reconstitution and diminished overall survival [1]. GVHD is the result of excessive alloreactivity occurring early after transplantation caused by donor T cells responding to antigens presented on recipient tissues. Under chronic stimulation, alloreactive T cells proliferate and release cytokines further stimulating immune responses to induce tissue damage. High-dose corticosteroids are the first-line treatment for acute GVHD, but are associated with a further delay of immune reconstitution, limited efficacy, and significant infection-related mortality [2]. Consequently, prevention of GVHD without immunosuppressive therapy would be a critical step forward in promoting rapid immune reconstitution and improving HCT outcomes.

Recent work identifying the unique metabolic profile of immune cells has created opportunities for designing new therapies. T cell metabolism integrates different activating signals, the availability of substrates, and the chronicity of the stimulation. As a result, different T cell subsets possess unique metabolic profiles [3]. After activation, effector T cells possess a glycolytic profile where pathways expand to support rapid cell growth. The metabolic profile of highly alloreactive and pathogenic T cells reflects robust stimulation in an inflammatory milieu depleted of anabolic substrates. Consequently, these cells are highly dependent on the catabolic pathways of oxidative phosphorylation (OXPHOS) and fatty acid oxidation (FAO) to support proliferation and cell survival [4]. As a result of substrate diversion to support increased anaerobic glycolysis, production of antioxidants is limited in alloreactive T cells [4]. Although this metabolic phenotype may promote cell growth and survival, the combination of increased ROS production and decreased antioxidants make alloreactive and pathogenic T cells very susceptible to oxidative stress. We designed a novel photosensitizer 2-Se-Cl that targets the unique metabolic profile and exploits the inherent weakness of pathogenic T cells by inhibiting OXPHOS and increasing oxidative stress. In 2016 we showed that ex-vivo application of 2-Se-Cl selectively eliminates highly alloreactive lymphocytes from the graft prior to HCT to prevent GVHD while maintaining primary antipathogen immunity in the complete MHC-mismatched setting [5] In this report we furthered our investigations to determine whether 2-Se-Cl promotes a curative graft-versus-leukemia (GVL) response essential for a successful transplantation.

The elimination of unwanted immune responses from the transplant graft also provides us with a unique opportunity to investigate the changes in cell composition during reconstitution. The regeneration of a fully competent immune system after transplant is a dynamic and complex interplay of different immune cells that compete for a variety of nutrients, ligands, and growth factors. For this reason, identifying the determinants of immune reconstitution from observations of individual cell populations is difficult, and at best, incomplete. To better understand these dynamic cell interactions, a machine learning-based approach coupled with high dimensional flow cytometry was employed to take simultaneous measurements of the immune cells that participated in successful immune reconstitution. Using a mouse model of GVHD, We found that photodepletion with 2-Se-Cl significantly altered donor lymphocytes to produce a polyfunctional product with high expression of PD-1 and CTLA-4, which was associated with tumor clearance, and long-term survival. In contrast, recipients of non-PD treated cells eventually developed lethal GVHD but early in reconstitution had an expansion of functionally exhausted T cells.

## Materials and methods

### Animals

All studies were approved by the Animal Care and Use Committee of Wake Forest University. Female BALB/c, C57BL/6, and C3H/HeJ mice (The Jackson Laboratory, Bar Harbor, ME) were 8–12 wk of age at the time of transplant. All mice were housed in a specific pathogen–free facility for the duration of the study. Mice were monitored daily after cell transplant. Animals were first anesthetized with isoflourane and then euthanized by cervical dislocation.

### Cell isolation and stimulation

Peripheral blood samples were obtained from mice via the optical sinus on day 15 after HCT. PBMCs were separated using Lymphoprep density gradient centrifugation (Stemcell Technologies, Cambridge, MA) and rested in RPMI 1640 (Life Technologies, Gaithersburg, MD) supplemented with 10% heat-inactivated FCS. To stimulate T cells, isolated PBMCs were cultured in RPMI 1640 with phorbol myristate acetate (PMA, 50 ng/ml) and Ionomycin (500 ng/ml) for 5 hours in the presence of GolgiPlug and GolgiStop.

### Photodepletion (PD)

For all PD experiments, cells were suspended in a photosensitizer-rich medium (7.5 × 10^−8^ M) for 20 min, followed by 30 min in a photosensitizer-free medium. Cells were then exposed to 5 J/cm^2^ of light (600 nM, 65-W equivalent LED) and 180 rotations per minute and washed twice to facilitate photosensitizer removal in RPMI 1640 supplemented with 10% heat-inactivated FCS.

### Bone marrow transplantation

A complete MHC Ag-mismatched (C57BL/6 (H2^b^) → Balb/c (H2^d^)) murine model of HCT was used [6]. To prepare the photodepleted (PD)-treated primed splenocytes, donor C57BL/6 splenocytes were co-cultured for 4 days with irradiated (20 Gy) BALB/c splenocytes using a Gas-permeable Rapid Expansion device (G-Rex, Wilson-Wolf, Minneapolis, MN), and then PD. On the day of HCT, recipient Balb/c mice were irradiated (8.5 Gy), and then received 10^7^ T cell-depleted (TCD) donor-derived bone marrow cells (<0.1% mature CD3^+^ T cells) together with 10^5^ recipient specific (A20, Balb/c-derived) leukemia cells engineered to express firefly luciferase, and 5 x10^6^ PD—treated cells (treatment group) or untreated (control group 1) primed splenocytes via tail vein injection. To evaluate for the retention of 3rd party responses, C3H/HeJ (H2^k^) mice were irradiated (9.5 Gy) and received a combination of 10^7^ TCD donor-derived bone marrow cells accompanied by 5 x10^6^ PD—treated cells [5, 7]. To compare the efficacy of our approach to post-transplant cyclophosphamide (PTCy) administered for GVHD prophylaxis, a group of mice received 10^7^ TCD bone marrow cells accompanied by 5 x 10^6^ primed splenocytes on day of HCT, followed by 50 mg/kg cyclophosphamide i.p. on days + 3 and + 4 after transplant (control group 2) [8, 9]. All mice were monitored for signs of GVHD according to an established grading system, and mice with severe GVHD (overall score > 5) were euthanized [10].

### Bioluminescence imaging

A20 leukemia/lymphoma cells (ATCC TIB-208, Manassas, VA) were transduced prior to HCT per manufacturer’s recommendations with pre-made luciferase lentiviral particles expressing firefly luciferase 3 gene under an inducible suCMV promoter with co-expression of a GFP marker (CMV-Luciferase-(firefly)-2A-GFP(Neo); GenTArget, Inc, San Diego, CA). GFP-expressing cells were then isolated by FACS to > 98% purity. To image tumor cells in vivo after HCT, mice were injected i.p. with D-Luciferin (Caliper LifeSciences, Hopkinton, MA) at a dose of 10 μL/g of body weight suspended in PBS. The animals were anesthetized with isoflurane (2% in 1 L/min oxygen), and bioluminescence images were acquired using the IVIS Lumina^®^ system (PerkinElmer, Hopkinton, MA). Images were analyzed and quantitated using Living Image software.

### Reagents for flow cytometry

The following monoclonal antibodies were used and purchased from BD Biosciences: hamster anti-mouse α-CD3-Cyanin-5-phycoerytherin (PE-Cy5; clone 145-2C11); α-CD279- Allophycocyanin-R700 (APC-R700, clone J43); α-CD152-PE-CF594 (clone UC10-4F10-11); rat anti-mouse α-CD4-Brilliant Ultraviolet-737 (BUV737; clone RM4-5); α-CD8-BUV395 (clone 53–6.7); α-CD44-Brilliant Blue-700 (BB700; clone IM7); TNF-α-Brilliant Violet-711 (BV711; clone MP6-XT22); α-CD25- Cyanin-7-phycoerytherin (PE-Cy7; clone PC61); α-CD62L-BV605 (clone MEL-14); IL-2-APC-Cy7 (clone JES6-5H4); α-CD223-BV421 (clone C9B7W); and mouse anti-mouse H2K^b^-BV786 (clone AF6-88.5); IFN-γ-BV650 (clone 4S.B3). Fixable Viability Stain 510 (FVS 510) was purchased from BD Biosciences and used to identify viable cells for FACS analysis. Surface staining was performed by incubation of Abs at a 1:100 dilution in fluorescence-activated cell sorter (FACS) buffer for 30 min on ice. To measure intracellular cytokine levels, cells were treated with the BD Biosciences Cytofix/Cytoperm kit according to the manufacturer’s instructions. Manual gating was performed on FlowJo software (TreeStar, San Francisco, CA).

### t-SNE dimensionality reduction of flow cytometry

Viable cells from all animals were identified by manual gating of PBMCs stained with antibodies and exported as flow cytometry standard (FCS) files from Flowjo and then imported into R. Samples were preprocessed to remove aggregates, labeled with animal and treatment, and subsequently, fluorescence channels were transformed by optimized Arcsinh and normalized by scaling [11]. Analysis of PBMC subsets was conducted in two steps. First, t-SNE was performed by using the RtSNE function from the Rtsne package to reduce the data to 2 dimensions. Second, the levels of each individual variable were calculated for each cell, and the z-score was determined. Using the ggplot2 package we plotted the V1 and V2 of t-SNE and the corresponding z-score for each variable.

### FlowSOM based analysis of T cells

Viable cells were identified by manual gating, and data were prepared and transformed as described above. SOM based unsupervised analysis was then performed using the FlowSOM R package [12][13]. By using the BuildSOM function, cells from surface and cytokine stains were assigned to a 6 × 5 grid. Cells in a pooled population were then separated by treatment group and mapped to the formed grid by using NewData function. Minimal Spanning Trees were built by using BuildMST function, so that the characteristics and the number of cells in each node could be visualized.

### Statistical analysis

Data from SOM node enumeration were analyzed by two way ANOVA with Neuman Keuls post test. Statistical tests were performed in InStat software (GraphPad Software, CA). A p-value of <0.05 was considered significant.

### Data sharing statement

For original data and R scripts please contact J.M.G. at jgrayson@wakehealth.edu.

## Results

### Photodepletion with 2-Se-Cl enriches for polyfunctional T cells with upregulation of inhibitory receptors PD-1 and CTLA-4

**Fig 1:**
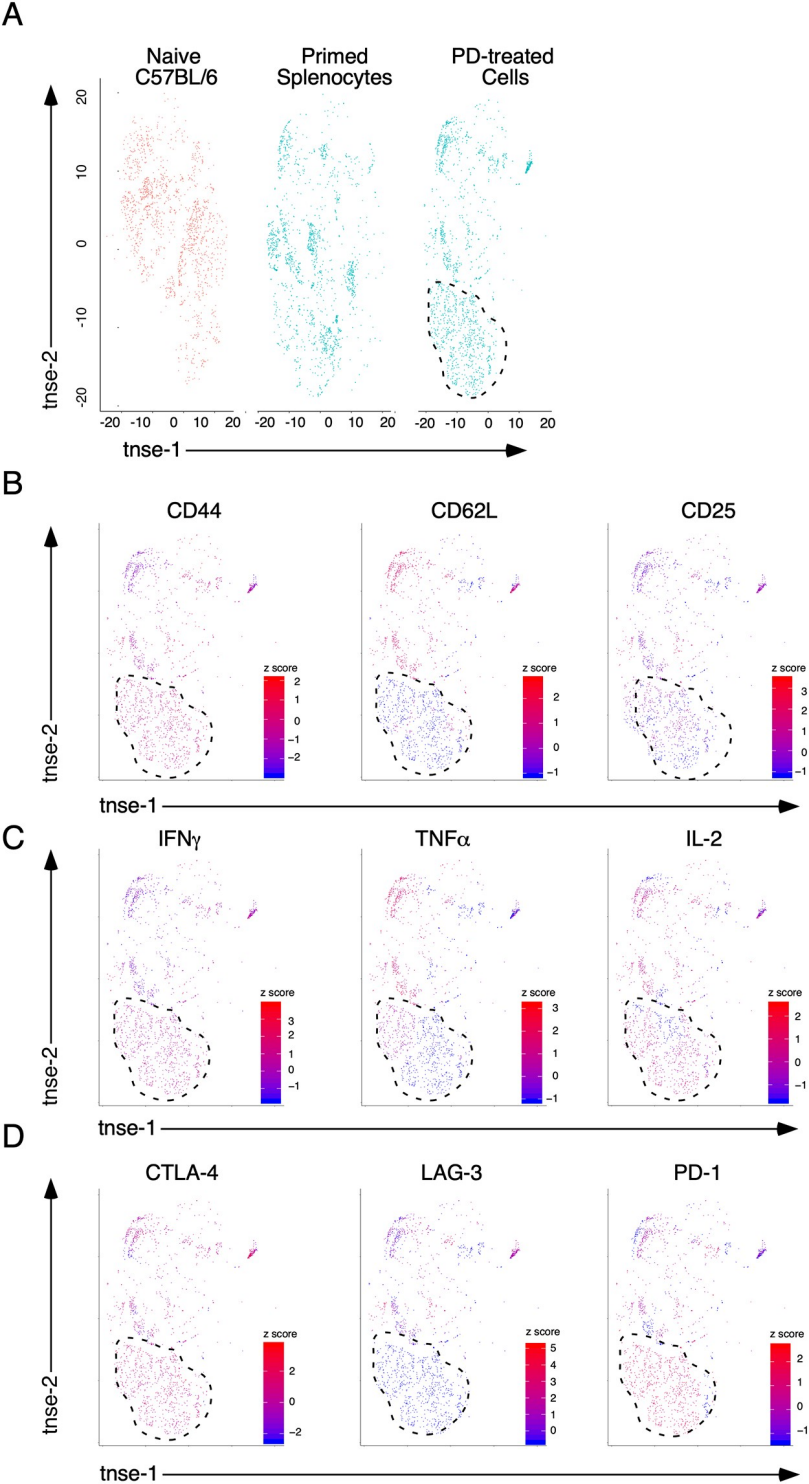
Photodepletion with 2-Se-Cl enriches polyfunctional T cells with upregulation of inhibitory receptors. (A) t-SNE transformation was performed on high-dimensional FACS data obtained from the analysis of isolated PBMCs from naïve C57BL/6 mice, and on primed splenocytes before and after PD. Area outlined by dotted line represents populations enriched by PD. Heat map analysis was performed of the individual variables mapped back to the z-score for each cell for (B) markers of effector status, (C) cytokine production, and (D) inhibitory receptor expression. Each group contains 9–15 mice in 3 independent experiments. Cells from multiple animals are included in each plot.

We have previously shown that ex-vivo application of 2- Se-Cl selectively eliminates activated highly alloreactive lymphocytes from the graft prior to HCT to prevent GVHD while maintaining antipathogen immunity [5]. The elimination of unwanted immune responses provides us with the opportunity to investigate the changes in cell composition that mediate clinical outcomes. For this purpose, a complete MHC Ag-mismatched (C57BL/6 (H2^b^) → Balb/c (H2^d^)) murine model of HCT was used. To investigate the effects of 2-Se-Cl treatment of primed splenocytes, donor C57BL/6 (H2^b^) splenocytes were cocultured for 4 days with irradiated (20 Gy) Balb/c (H2^d^) splenocytes, and then photodepleted (PD) with 2-Se-Cl. Multiparameter flow cytometry of cytokines and surface markers was performed on the primed splenocyte product before and 18 hours after photodepletion, and dimensionality reduction using t-stochastic neighbor embedding (t-SNE) was employed [14]. After transformation by t-SNE, unique immune signatures were observed (Fig 1A). Specifically, PD-treated cells were enriched for populations that were minimally present in primed splenocytes, and near absent in naïve C57BL/6 splenocytes (Fig 1A, area outlined). To further define populations enriched by PD, we performed a heat map analysis of the individual variables mapped back to the z-score for each cell. Analysis of the population enriched by PD revealed high CD44 expression, low expression of CD62L, and low or mixed expression of CD25 (Fig 1B). PD enriched cell populations displayed homogenous production of IFNγ, but heterogeneous production of TNFα and IL-2 (Fig 1C) following PMA and ION stimulation. Evaluation of inhibitory receptors revealed both CTLA-4 and PD-1 were upregulated at high levels on PD-enriched cells, without LAG-3 expression (Fig 1D). Taken together these results demonstrate that PD with 2-Se-Cl significantly alters Balb/c-primed, C57BL/6-splenocytes to produce a polyfunctional product, but with high expression of PD-1 and CTLA-4.

### Photodepletion with 2-Se-Cl prevents GVHD while maintaining antitumor immunity

**Fig 2:**
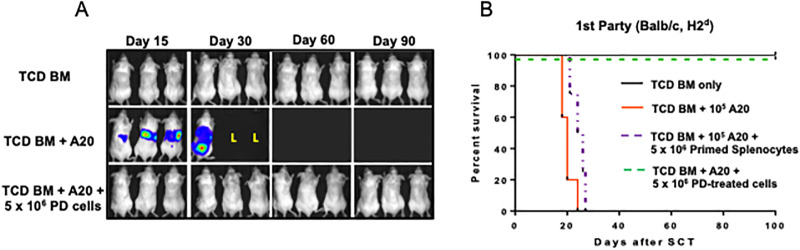
Photodepletion prevents GVHD while maintaining antitumor immunity. Donor C57BL/6 (H2^b^) splenocytes were cocultured for 4 days with irradiated (20 Gy) Balb/c (H2^d^) splenocytes, and then photodepleted (PD). On the day of HCT, 10^5^ luciferase-expressing A20 leukemia cells engineered to express firefly luciferase were infused into irradiated 1^st^-party BALB/c with 10^7^ donor C57BL/6 T-cell–depleted bone marrow cells (TCD BM). (A) Leukemic burden was measured weekly by bioluminescent imaging. On the day of HCT, 5 x 10^6^ C57BL/6 PD-treated or non-treated primed splenocytes were infused into lethally irradiated (B) BALB/c (1st-party) mice. Three mice/group underwent HCT in 3–5 independent experiments in each group.

To verify retention of antitumor immunity following exposure to 2-Se-Cl, C57BL/6 PD-treated splenocytes were infused into lethally irradiated BALB/c (first-party) along with 10^5^ Balb/c-derived A20 leukemia cells engineered to express firefly luciferase, and 10^7^ TCD C57BL/6-derived bone marrow cells. Leukemic burden was measured weekly by bioluminescent imaging (Fig 2A) and compared to controls (recipients of TCD BM + A20 only). Balb/c recipient mice that also received PD-treated splenocytes survived > 100 days without evidence of GVHD or leukemia. In contrast, all mice inoculated with leukemia at the time of HCT without PD-treated splenocytes died of leukemia progression (mean of 21 days). Additionally, mice that received untreated primed splenocytes died from GVHD progression (mean of 25 days, Fig 2B). These animals had background levels of fluorescence demonstrating no leukemic burden and elevated GVHD scores (S1 Fig) We next evaluated the effects of standard-of-care prophylaxis in our model for comparison and found that Balb/c recipient mice that received primed splenocytes at time of transplant followed by post-transplant cyclophosphamide (PTCy) at a clinically relevant dose (50mg/kg) on days 3 and 4 after transplant died of lethal GVHD (mean 37 days). These results demonstrate that PD of activated splenocytes with 2-Se-Cl prevents lethal GVHD while preserving antitumor immunity in an aggressive model of disease better than current standard of care.

### HCT outcomes are associated with distinct immune signatures early after transplant

**Fig 3:**
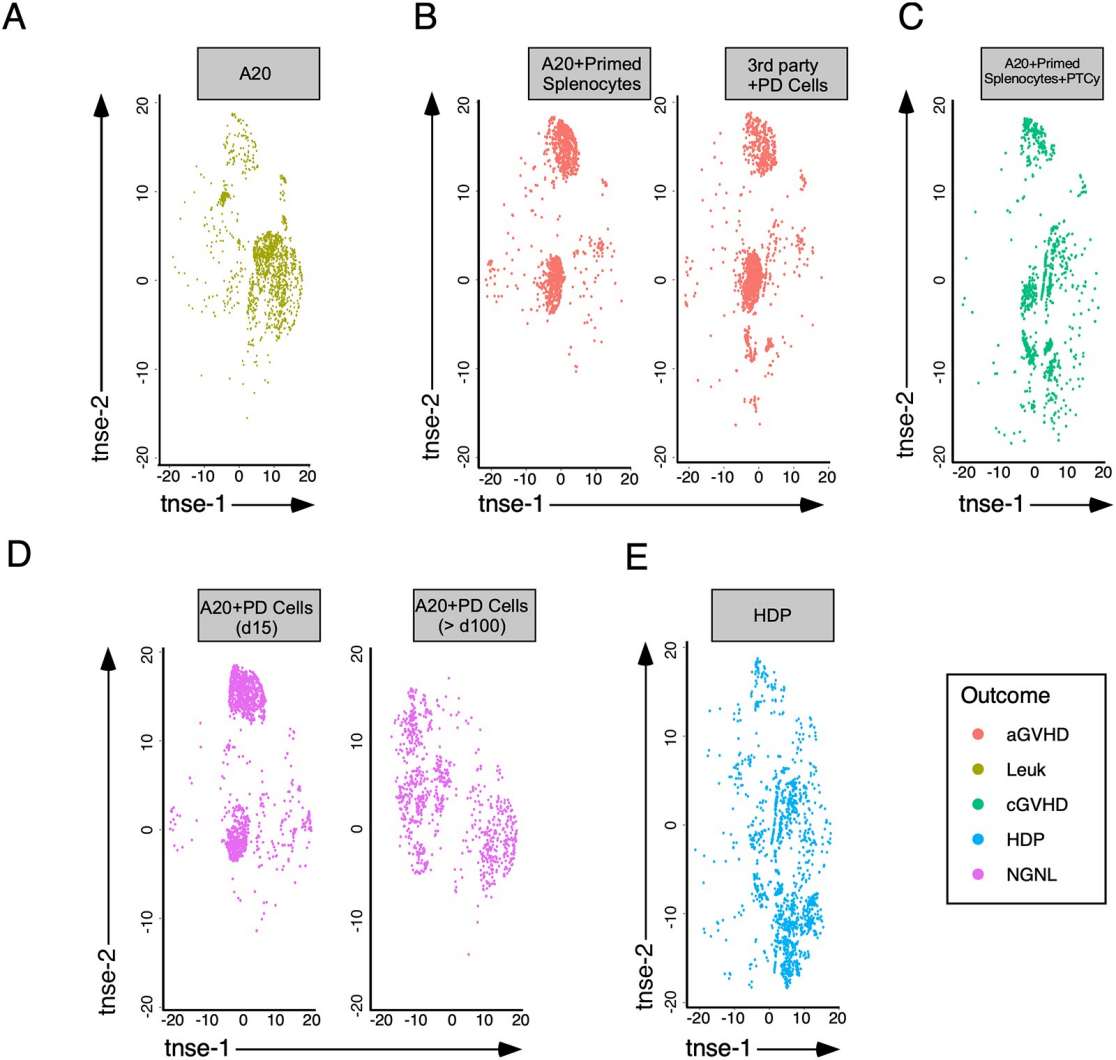
GVHD and antitumor immunity are associated with unique immune signatures early after transplant. t-SNE transformation was performed on the high-dimensional FACS data obtained from the analysis of isolated PBMCs from recipient mice 15 or 100 days after HCT. The immune signatures are shown for mice that received (A) A20 leukemia alone, (B) A20 + primed splenocytes, or for 3^rd^- party recipients of PD-treated cells, (C) A20 + primed splenocytes + PTCy, and (D) A20 + PD-treated cells (Days 15 and 100). (E) As a control for homeostatic driven proliferation (HDP) in a lymphopenic environment, irradiated C57BL/6 mice received PD-treated cells. aGVHD, acute GVHD; cGVHD, predominantly cutaneous GVHD; Leuk, died of leukemia progression without GVHD; HDP, homeostatic driven proliferation; NGNL; long-term survival with no GVHD and no leukemia. Each group contains 9–15 mice in 3 independent experiments. Cells from multiple animals are included in each plot.

To investigate the effects of early immune reconstitution on transplant outcomes, we sampled peripheral blood from all groups of mice 15 days after HCT. FACS analysis was performed on isolated PBMCs for enumeration and function, and dimensionality reduction by t-SNE was again employed on the data. After reduction by t-SNE, unique immune signatures were observed (Fig 3, S2–S6 Figs). Animals that only received TCD BM and A20 leukemia died at a mean of 21 days after transplant from leukemia progression, and had increased cells with high tsne-1 and low tsne-2 values (Fig 3A). In contrast, immune signatures of 1^st^-party mice that received primed-splenocytes and 3^rd^-party mice (C57BL/6 PD-treated BALC/c primed splenocytes infused into lethally irradiated C3H/HeJ (H2^k^)) that received PD-treated cells, both which developed lethal GVHD had low tsne-1 and variable tsne-2 values. The two discrete populations of cells were CD3^+^ (S2 Fig) and were either CD4^+^ (upper, S3 Fig) or CD8^+^ (center, S4 Fig). Both of these populations were positive for PD-1 and LAG-3 (S5 and S6 Figs). Since PTCy has been highly successful in preventing GVHD, we chose to evaluate the effects of PTCy on early immune reconstitution in our model (Fig 3C). At day 15 after transplant, mice that received PTCy, had a decrease in the enriched populations observed in 1st and 3^rd^ party transplant. In comparison, recipients of PD-treated cells initially were similar to 1^st^ and 3^rd^ party transfers (Fig 3D), but by 100 days after transplant the cell populations of 1^st^-party PD recipients evolved significantly and appeared similar to those of naïve C57BL/6 splenocytes (Fig 1A). To examine the effect of homeostatic driven proliferation (HDP) in a lymphopenic environment, we transferred PD-treated cells into irradiated C57BL/6 mice. Fifteen days later the populations in the lower right of the t-SNE plot predominated similar to the results observed with PTCy treatment (Fig 3E). Taken together, these results demonstrate that high dimensional flow cytometry analysis reveals a diverse landscape across different outcomes following transplantation.

### Successful generation of antitumor immunity is associated with an expansion of polyfunctional T cells and monocyte-derived DCs early after transplant

**Fig 4:**
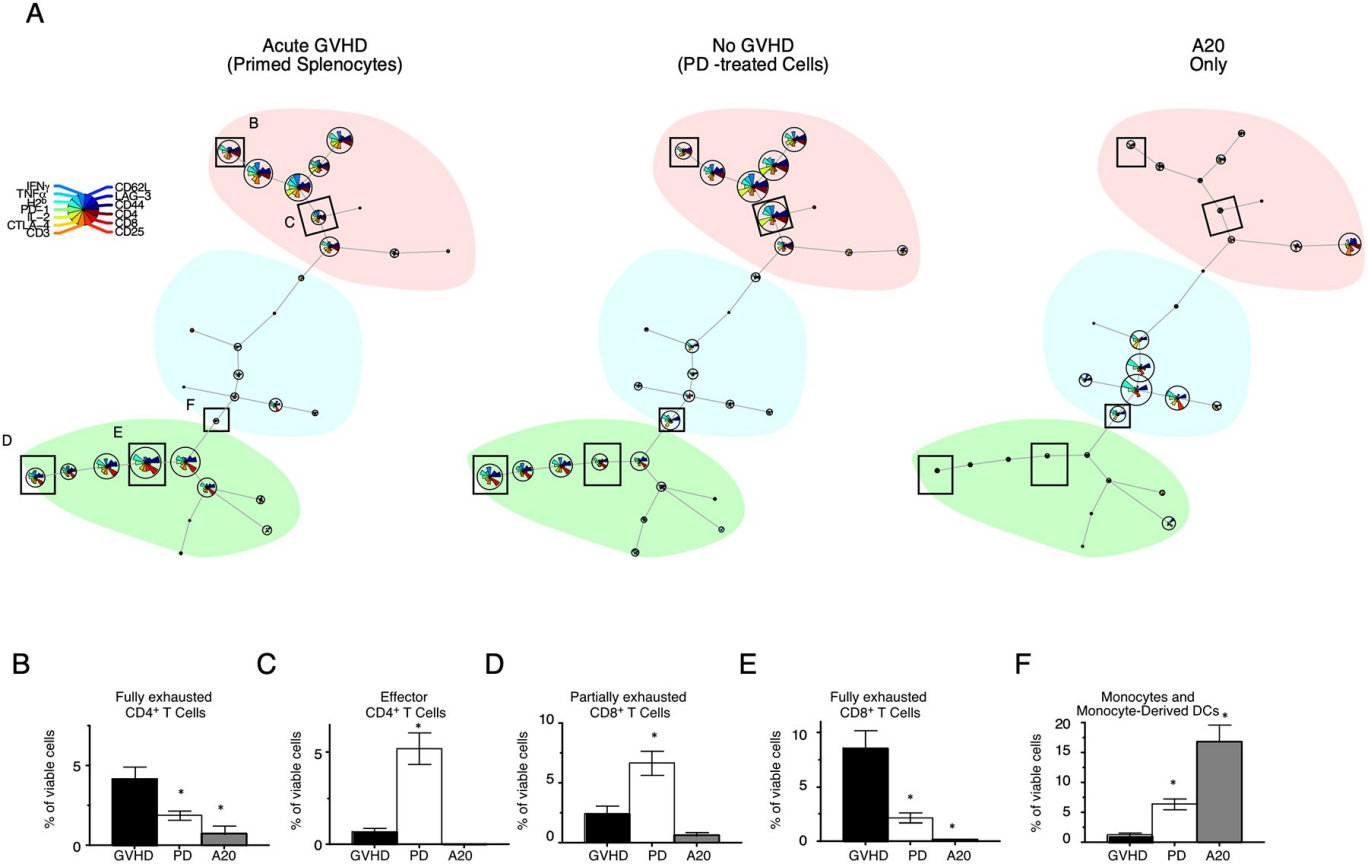
Antitumor immunity is associated with an expansion of polyfunctional T cells and monocyte-derived DCs early after transplant. (A) SOM transformation was performed on high-dimensional FACS data obtained from the analysis of isolated PBMCs collected 15 days after transplant from mice that received primed splenocytes or PD-treated cells at time of transplantation. SOM nodes that were different between treatments were identified and outlined in rectangles. (B-F) Subpopulations of T cells were identified by the phenotype represented in each pair of discordant nodes, and labeled according to functional status. Bar graphs represent quantification of subpopulations identified in pairs of discordant nodes. Mean ± SEM are plotted. * p-value < 0.01. GVHD, recipients of primed splenocytes that died of lethal GVHD; PD, recipients of PD-treated cells that lived long term. Each group contains 9–15 mice in 3 independent experiments.

Transformation of data by t-SNE facilitates visualization of differences in a high dimensional space. However, self-organizing map (SOM) analysis can be applied to identify and quantitate multivariable differences in subpopulations of cells [13, 15]. SOMs produced from PBMCs isolated from mice 15 days after transplant that had received primed splenocytes with or without PD were mapped to a network of 30 nodes broadly organized into CD4^+^, CD8^+^, and CD3^-^ cell populations (Fig 4). Nodes that were different between treatments were identified and outlined in rectangles in Fig 4A. Quantification of nodes between maps revealed significant differences in the subpopulations of cells in mice that developed acute GVHD (Fig 4B–4F). Subpopulations of T cells were identified by the phenotype represented in each pair of discordant nodes, and labeled according to functional status as assessed by cytokine production as well as expression of inhibitory receptors. Mice that developed lethal GVHD died a median of 21 days after transplant, and had a marked increase in exhausted T cells [16–18]. CD4^+^ and CD8^+^ T cells from these mice had high surface expression of CD44 and CD25, coexpression of PD-1 and CTLA-4, and lacked detectable cytokine production (Fig 4B and 4E) following stimulation. In contrast recipients of PD-splenocytes, that survived long-term without GVHD, had expansions of polyfunctional CD4^+^T cells (CD44^hi^IFNγ^+^TNFα^+^IL-2^+^) that lacked inhibitory receptor expression, and partially exhausted CD8^+^ T cells (CD44^hi^IFNγ^+^TNFα^low^) with up-regulated PD-1, CTLA-4, and LAG-3 (Fig 4C and 4D). In the non-T cell compartment, recipients of PD-treated cells had an expansion of CD3^-^CD44^hi^ cells with up-regulated CTLA-4 early after transplant, likely representing expansion of monocytes and monocyte-derived dendritic cells (DCs) (Fig 4F) [19–21]. These results demonstrate that GVHD was associated with a distinct immune signature before disease onset (day 15) that was marked by an expansion of exhausted T cells. In contrast, generation of antitumor immunity and long-term survival was associated with an expansion of polyfunctional T cells, monocytes, and DCs early after transplantation.

## Discussion

The photosensitizer 2-Se-Cl targets the unique metabolic profile and exploits the inherent weakness of alloreactive T cells by inhibiting oxidative phosphorylation and increasing oxidative stress. We have previously shown that after priming, alloreactive T cells are highly oxidative, and that 2-Se-Cl can be employed ex-vivo to prevent GVHD while maintaining primary antiviral immunity [5]. In this report, we demonstrate PD with 2-Se-Cl also preserves curative antitumor immunity. By combining the application of this therapeutic with high dimensional flow cytometry performed on peripheral blood samples collected 15 days after HCT, we uncovered patterns of immune reconstitution, and identified unique immune signatures associated with GVHD and graft-versus-leukemia immunity. In our model, the early stages of GVHD prior to overt disease were marked by an expansion of functionally exhausted T cells, while successful antitumor immunity was associated with an expansion of polyfunctional T cells, monocytes, and DCs early after transplantation.

The elimination of pathogenic immune responses coupled with a machine learning analysis of early reconstituting T cells revealed unique differences associated with HCT outcomes. The use of high-dimensional FACS analysis allowed us to make simultaneous measurements of a multitude of immune cells actively participating in the regeneration of immune competence. However, efficiently analyzing high-dimensional FACS data by traditional methods requires basic assumptions that may bias results. Alternatively, the transformation of the high-dimensional data into low-dimensional space by unsupervised machine learning approaches adapts independently to the data by iteration, and therefore minimizes bias. In our study, initial transformation of the data by t-SNE reveled differences in a high dimensional space between immune signatures. These differences were clearly seen in mice that did not receive T cells at the time of transplant and PTCy–treated animals compared to other groups, with absence of effector T cell populations that localized along the t-SNE-2 axis. However, animals that received PD or non-PD primed splenocytes shared similar t-SNE signatures not easily distinguished from each other, with prominent enrichment of cell populations along the t-SNE-2 axis. Transformation of the data by SOM analysis clustered cells by similarities in surface phenotype and cytokine production to improve resolution. After SOM transformation, unique population differences were evident between mice that developed lethal GVHD and recipients of PD-treated splenocytes that did not. SOM transformation revealed a prominence of polyfunctional effector CD4^+^ T cells in mice that received a PD-treated product at time of transplant, with only partially exhausted CD8^+^ T cells co-expressing inhibitory receptors. In contrast, the immune signatures detected by SOM transformation from mice that received primed splenocytes and died of lethal GVHD, identified expansions of exhausted CD4^+^ and CD8^+^ T cells before disease onset.

For many years investigators have developed approaches to allow mismatched HCT to increase the availability of potentially curative modality to treat cancer, autoimmunity, and immunodeficiencies. Various strategies including conjugation of toxins to CD25 [22], depletion of naïve phenotype T cells [23] and early versions of photodepletion [6] have been used in preclinical and clinical studies. But to date none have become standard of care. This prompted us to design an agent that would selectively target alloreactive cells based on bioenergetics. The success of our approach in preventing GVHD while maintaining antitumor immunity is most likely the result of the elimination of dominant alloreactive cells coupled with the induced expression of inhibitory receptors on CD8^+^ T cells. The removal of the most alloreactive cells by 2-Se-Cl may have permitted the engagement of PD-1 and CTLA-4 receptors by ligands expressed on recipient tissues to further prevent GVHD and promote the rapid expansion of non-pathogenic cells with antileukemic potential [24, 25]. This hypothesis is supported by the increase in polyfunctional CD4^+^ T cells and only partial exhaustion of the CD8^+^ T cells noted 15 days after transplant in recipients of PD-treated splenocytes. T cell exhaustion is characterized by the stepwise loss of function [17]. Factors that determine the severity include the duration and magnitude of antigenic activation, availability of CD4^+^ T cell help, and the presence of stimulatory and suppressive cytokines and receptors [26]. Consequently, modulation of these factors may further promote exhaustion, or reverse the exhaustion and allow cells to regain polyfunctionality. Accordingly, in our model, the state of partial exhaustion of the CD8^+^ T cells may represent less alloreactive cells with the potential to cause GVHD or T cells responding to unique antigens present on the leukemia. Therefore, the increase in polyfunctional CD4^+^ T cells without the manifestations of GVHD may reflect the development of immune tolerance that inhibited the expansion of pathogenic T cells, or the development of sufficient CD4^+^ T cell support that facilitated the rapid clearance of leukemia, or both. Taken together, these changes seen in the immune signatures 15 days after transplant of mice that received PD-treated cells portray a dynamic interplay between T cells that facilitated the successful development of antitumor immunity and prevention of GVHD.

Employing 2-Se-Cl is superior to the application of PTCy for GVHD prophylaxis in our model. However, PTCy is highly effective clinically in preventing GVHD, and has revolutionized the field of HCT by effectively reducing the risk of GVHD associated with haploidentical transplant to that of HLA-matched related and unrelated donor transplantations [27–29]. Why then did PTCy fail to prevent GVHD in our model? The answer most likely is due to the infusion of primed splenocytes at the time of transplant. In this setting, already primed splenocytes may have the advantage and initiated tissue damage immediately upon infusion. The subsequent rapid expansion and release of stimulatory cytokines may have promoted the sequestration of pathogenic T cells in targeted organs in the initial days following infusion enhancing their resistance to PTCy administration. Interestingly, all animals that received PTCy died of severe cutaneous GVHD with a mean of 37 days after HCT, indicating a kinetic benefit of PTCy administration compared to recipients of primed splenocytes that did not receive PTCy (mean 25 day survival), and a skewing of the GVHD response. This outcome was also associated with an immune signature early after transplant that was enriched with T cells that did not express the inhibitory receptors PD-1, CTLA-4, or LAG-3. Although PTCy administration failed to prevent GVHD in our model, its effects on the early events of immune reconstitution were profound, and revealed an immune signature that may be associated with aggressive cutaneous GVHD that is worthy of further investigation.

Extensive investigations have been conducted to identify biomarkers associated with acute GVHD with the ultimate goal of risk stratification to facilitate timely therapeutic intervention [30]. However, biomarkers with sufficient sensitivity to predict GVHD and facilitate preemptive therapy are limited, and need further development [30, 31]. We chose a time point early after transplant and before manifestations of GVHD to identify changes that may provide insights into the pathophysiology of the disease. On day 15 after transplant, mice that eventually developed lethal GVHD had a significant expansion of completely exhausted CD4^+^ and CD8^+^ T cells marked by lack of cytokine production following stimulation. T cell exhaustion also occurs in other disease settings such as in chronic infection and cancer, and may serve as an early indicator of disease relapse [16, 32–36].

One limitation of our study is that we chose to restrict our investigations to a single model of lethal GVHD with an aggressive leukemia. This choice was made to minimize variations within groups, which facilitated the identification of unique immune signatures associated the difference outcomes. We anticipate that the degree and quality of the immune responses may vary by mouse strain, MHC disparity, and type of malignancy. Additionally, employing murine model systems can only estimate immunity in humans, and is unable to account for environmental influences unique to humans. Although our model has allowed us identify distinct immune signatures associated with HCT outcomes, additional studies in humans are required to confirm our results. We are currently conducting similar investigations on clinical samples for this purpose.

In conclusion, we employed the novel photosensitizer 2-Se-Cl to prevent lethal GVHD while maintaining antitumor immunity in an aggressive murine model of the disease. We then conducted a machine learning-based approach to take simultaneous measurements of reconstituting immune cells in the early post-transplant period, and uncovered distinct immune signatures associated with GVHD and graft-versus-leukemia immunity in our model. Specifically, GVHD was marked by an expansion of exhausted T cells in the blood before disease onset, while successful antitumor immunity was associated with an expansion of polyfunctional T cells, monocytes, and DCs early after transplantation. Future applications of our approach are broad, and include preventing GVHD in multiple transplant settings, and employing machine learning techniques to enrich the development of biomarkers with the goal of improving predictive models of HCT outcomes and opportunities for therapeutic interventions.

## Supporting information

S1 Fig A20 bearing recipients receiving primed splenocytes eliminate leukemia but develop severe GVHD. Donor C57BL/6 (H2^b^) splenocytes were cocultured for 4 days with irradiated (20 Gy) Balb/c (H2^d^) splenocytes. On the day of HCT, 10^5^ luciferase-expressing A20 leukemia cells engineered to express firefly luciferase were infused into irradiated 1^st^-party BALB/c with 10^7^ donor C57BL/6 T-cell–depleted bone marrow cells (TCD BM) and 5 x 10^6^ C57BL/6 non-treated primed splenocytes. (A) Leukemic burden was quantitated on day 15 by measuring maximum photon counts on images collected from mice. (B) GHVD scores are plotted for the indicated recipients. Three mice/group underwent HCT in 3–5 independent experiments in each group. A representative experiment is shown. Average and standard deviation are plotted. * p-value < 0.01.
(TIF)

S2 Fig GVHD and antitumor immunity are associated with changes in CD3^+^ T cells early after transplant. t-SNE transformation was performed on the high-dimensional FACS data obtained from the analysis of isolated PBMCs from recipient mice 15 or 100 days after HCT. The immune signatures are shown for mice that received (A) A20 leukemia alone, (B) A20 + primed splenocytes, or for 3^rd^- party recipients of PD-treated cells, (C) A20 + primed splenocytes + PTCy, and (D) A20 + PD-treated cells (Days 15 and 100). (E) As a control for homeostatic driven proliferation (HDP) in a lymphopenic environment, irradiated C57BL/6 mice received PD-treated cells. Heat map analysis was performed for CD3 and mapped back to the z-score for each cell. Each group contains 9–15 mice in 3 independent experiments. Cells from multiple animals are included in each plot.
(TIF)

S3 Fig GVHD and antitumor immunity are associated with changes in CD4^+^ T cells early after transplant. t-SNE transformation was performed on the high-dimensional FACS data obtained from the analysis of isolated PBMCs from recipient mice 15 or 100 days after HCT. The immune signatures are shown for mice that received (A) A20 leukemia alone, (B) A20 + primed splenocytes, or for 3^rd^- party recipients of PD-treated cells, (C) A20 + primed splenocytes + PTCy, and (D) A20 + PD-treated cells (Days 15 and 100). (E) As a control for homeostatic driven proliferation (HDP) in a lymphopenic environment, irradiated C57BL/6 mice received PD-treated cells. Heat map analysis was performed for CD4 and mapped back to the z-score for each cell. Each group contains 9–15 mice in 3 independent experiments. Cells from multiple animals are included in each plot.
(TIF)

S4 Fig GVHD and antitumor immunity are associated with changes in CD8^+^ T cells early after transplant. t-SNE transformation was performed on the high-dimensional FACS data obtained from the analysis of isolated PBMCs from recipient mice 15 or 100 days after HCT. The immune signatures are shown for mice that received (A) A20 leukemia alone, (B) A20 + primed splenocytes, or for 3^rd^- party recipients of PD-treated cells, (C) A20 + primed splenocytes + PTCy, and (D) A20 + PD-treated cells (Days 15 and 100). (E) As a control for homeostatic driven proliferation (HDP) in a lymphopenic environment, irradiated C57BL/6 mice received PD-treated cells. Heat map analysis was performed for CD8 and mapped back to the z-score for each cell. Each group contains 9–15 mice in 3 independent experiments. Cells from multiple animals are included in each plot.
(TIF)

S5 Fig GVHD and antitumor immunity are associated with changes in LAG-3^+^ cells early after transplant. t-SNE transformation was performed on the high-dimensional FACS data obtained from the analysis of isolated PBMCs from recipient mice 15 or 100 days after HCT. The immune signatures are shown for mice that received (A) A20 leukemia alone, (B) A20 + primed splenocytes, or for 3^rd^- party recipients of PD-treated cells, (C) A20 + primed splenocytes + PTCy, and (D) A20 + PD-treated cells (Days 15 and 100). (E) As a control for homeostatic driven proliferation (HDP) in a lymphopenic environment, irradiated C57BL/6 mice received PD-treated cells. Heat map analysis was performed for LAG-3 and mapped back to the z-score for each cell. Each group contains 9–15 mice in 3 independent experiments. Cells from multiple animals are included in each plot.
(TIF)

S6 Fig GVHD and antitumor immunity are associated with changes in PD-1^+^ cells early after transplant. t-SNE transformation was performed on the high-dimensional FACS data obtained from the analysis of isolated PBMCs from recipient mice 15 or 100 days after HCT. The immune signatures are shown for mice that received (A) A20 leukemia alone, (B) A20 + primed splenocytes, or for 3^rd^- party recipients of PD-treated cells, (C) A20 + primed splenocytes + PTCy, and (D) A20 + PD-treated cells (Days 15 and 100). (E) As a control for homeostatic driven proliferation (HDP) in a lymphopenic environment, irradiated C57BL/6 mice received PD-treated cells. Heat map analysis was performed for PD-1 and mapped back to the z-score for each cell. Each group contains 9–15 mice in 3 independent experiments. Cells from multiple animals are included in each plot.
(TIF)

S1 Data The arrive guidelines. (PDF)
