# Supplementary figures and images for: Photodepletion with 2-Se-Cl prevents lethal graft-versus-host disease while preserving antitumor immunity
Source: PLoS One. 2020 Jun 22;15(6):e0234778. doi: 10.1371/journal.pone.0234778 (PMC7307732; doi:10.1371/journal.pone.0234778)

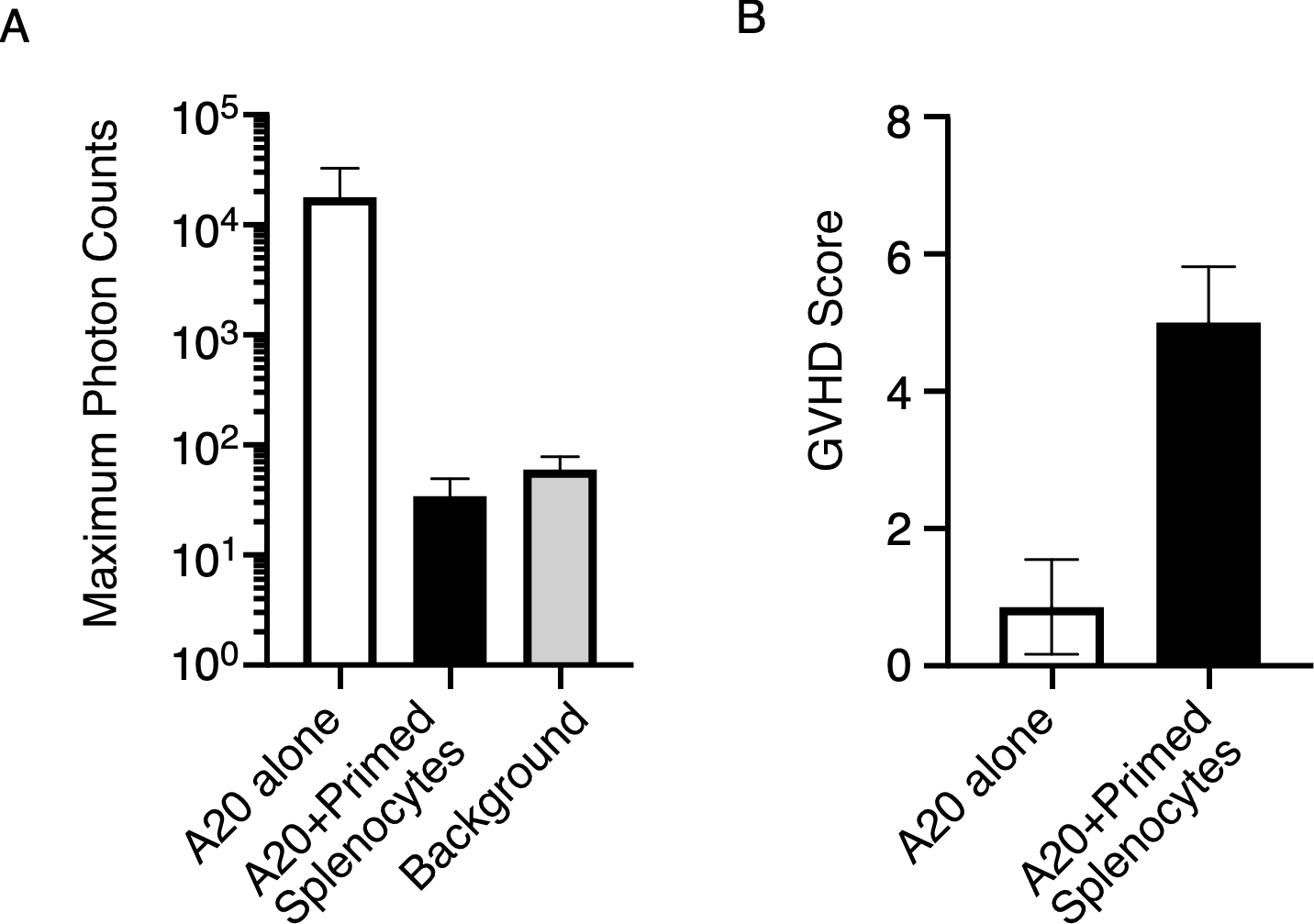

Supplement: S1 Fig — Donor C57BL/6 (H2b) splenocytes were cocultured for 4 days with irradiated (20 Gy) Balb/c (H2d) splenocytes. On the day of HCT, 105 luciferase-expressing A20 leukemia cells engineered to express firefly luciferase were infused into irradiated 1st-party BALB/c with 107 donor C57BL/6 T-cell–depleted bone marrow cells (TCD BM) and 5 x 10^6^ C57BL/6 non-treated primed splenocytes. (A) Leukemic burden was quantitated on day 15 by measuring maximum photon counts on images collected from mice. (B) GHVD scores are plotted for the indicated recipients. Three mice/group underwent HCT in 3–5 independent experiments in each group. A representative experiment is shown. Average and standard deviation are plotted. * p-value < 0.01. (TIF) [file pone.0234778.s001.tif]

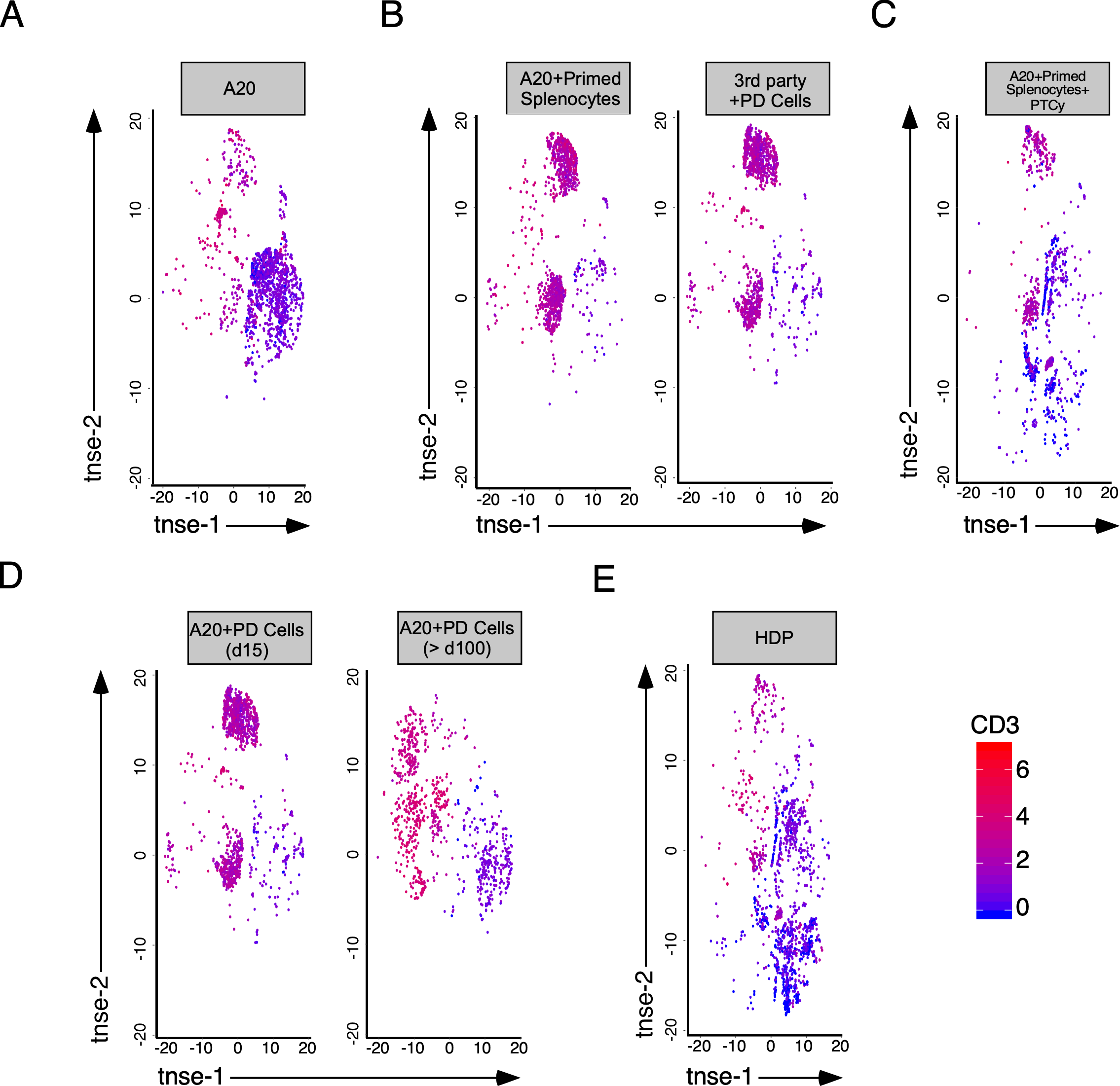

Supplement: S2 Fig — t-SNE transformation was performed on the high-dimensional FACS data obtained from the analysis of isolated PBMCs from recipient mice 15 or 100 days after HCT. The immune signatures are shown for mice that received (A) A20 leukemia alone, (B) A20 + primed splenocytes, or for 3rd- party recipients of PD-treated cells, (C) A20 + primed splenocytes + PTCy, and (D) A20 + PD-treated cells (Days 15 and 100). (E) As a control for homeostatic driven proliferation (HDP) in a lymphopenic environment, irradiated C57BL/6 mice received PD-treated cells. Heat map analysis was performed for CD3 and mapped back to the z-score for each cell. Each group contains 9–15 mice in 3 independent experiments. Cells from multiple animals are included in each plot. (TIF) [file pone.0234778.s002.tif]

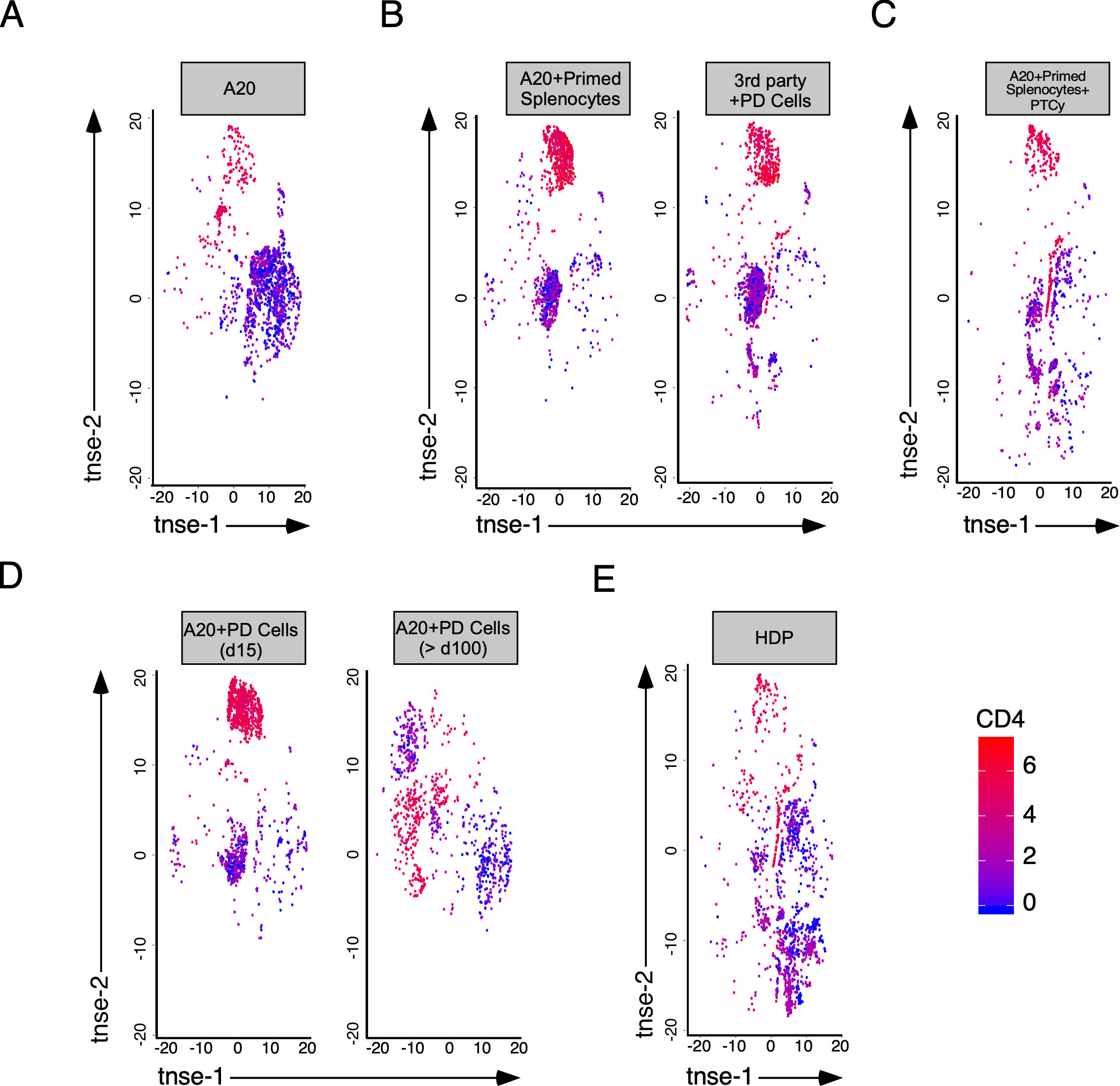

Supplement: S3 Fig — t-SNE transformation was performed on the high-dimensional FACS data obtained from the analysis of isolated PBMCs from recipient mice 15 or 100 days after HCT. The immune signatures are shown for mice that received (A) A20 leukemia alone, (B) A20 + primed splenocytes, or for 3rd- party recipients of PD-treated cells, (C) A20 + primed splenocytes + PTCy, and (D) A20 + PD-treated cells (Days 15 and 100). (E) As a control for homeostatic driven proliferation (HDP) in a lymphopenic environment, irradiated C57BL/6 mice received PD-treated cells. Heat map analysis was performed for CD4 and mapped back to the z-score for each cell. Each group contains 9–15 mice in 3 independent experiments. Cells from multiple animals are included in each plot. (TIF) [file pone.0234778.s003.tif]

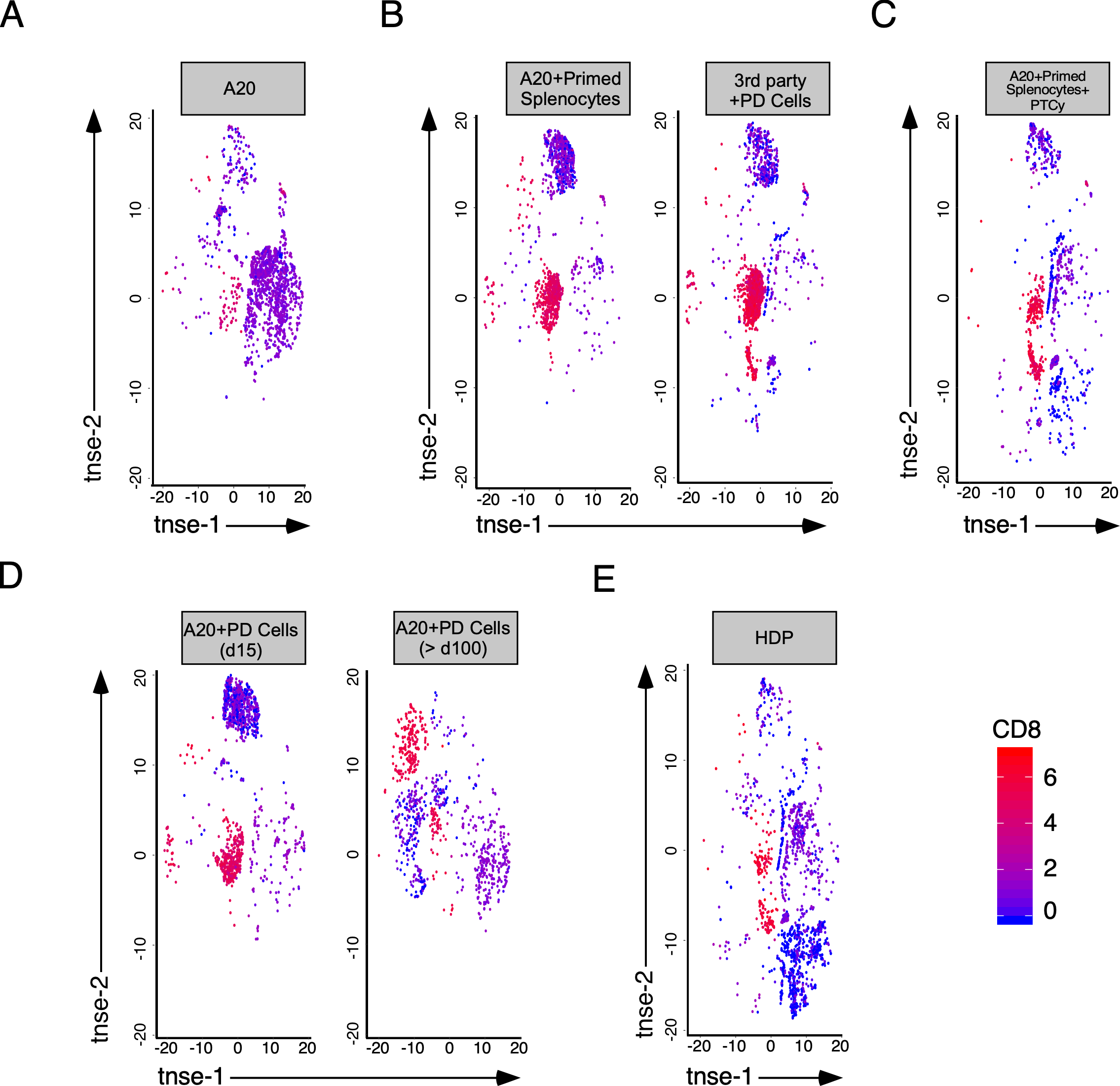

Supplement: S4 Fig — t-SNE transformation was performed on the high-dimensional FACS data obtained from the analysis of isolated PBMCs from recipient mice 15 or 100 days after HCT. The immune signatures are shown for mice that received (A) A20 leukemia alone, (B) A20 + primed splenocytes, or for 3rd- party recipients of PD-treated cells, (C) A20 + primed splenocytes + PTCy, and (D) A20 + PD-treated cells (Days 15 and 100). (E) As a control for homeostatic driven proliferation (HDP) in a lymphopenic environment, irradiated C57BL/6 mice received PD-treated cells. Heat map analysis was performed for CD8 and mapped back to the z-score for each cell. Each group contains 9–15 mice in 3 independent experiments. Cells from multiple animals are included in each plot. (TIF) [file pone.0234778.s004.tif]

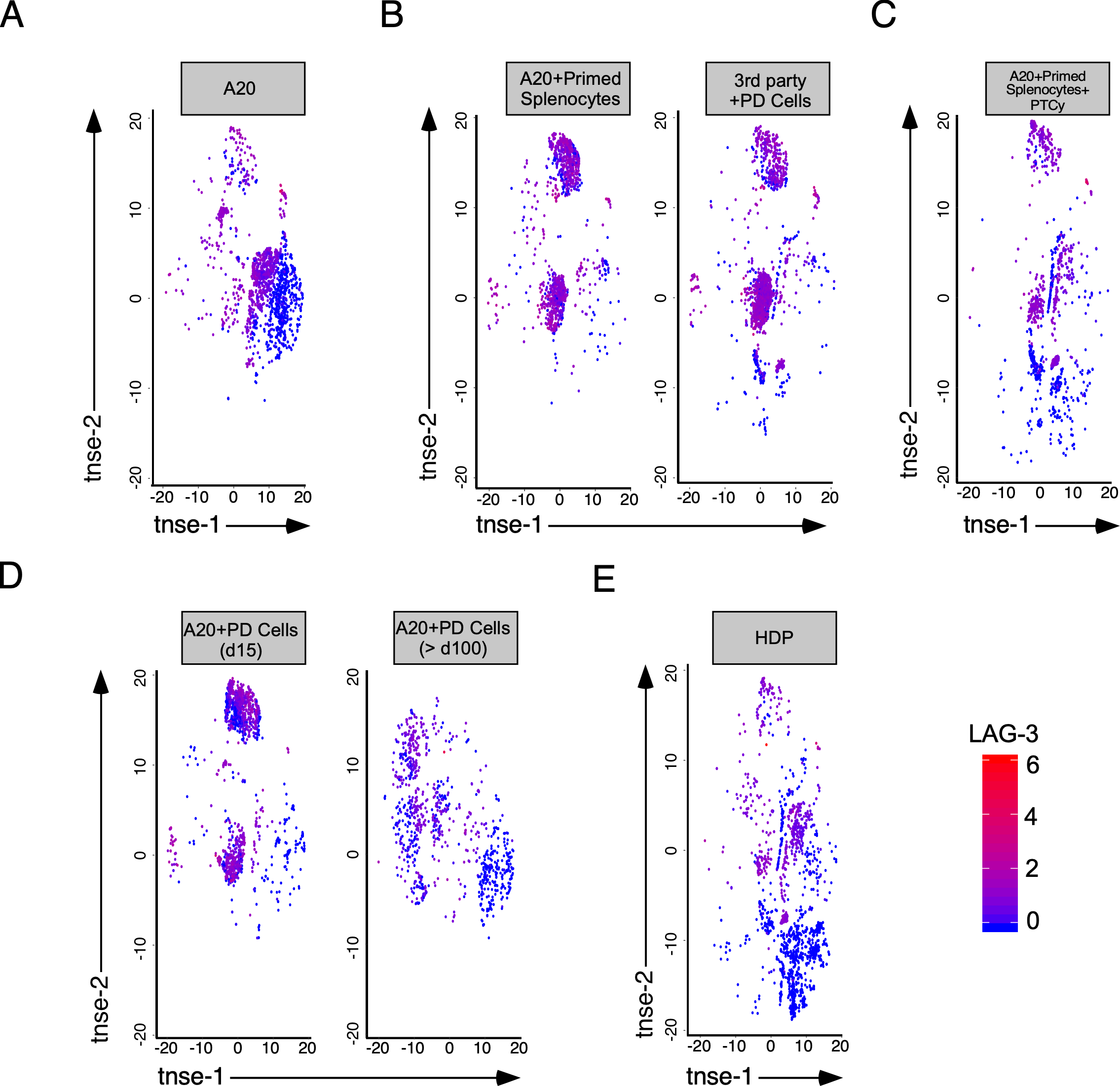

Supplement: S5 Fig — t-SNE transformation was performed on the high-dimensional FACS data obtained from the analysis of isolated PBMCs from recipient mice 15 or 100 days after HCT. The immune signatures are shown for mice that received (A) A20 leukemia alone, (B) A20 + primed splenocytes, or for 3rd- party recipients of PD-treated cells, (C) A20 + primed splenocytes + PTCy, and (D) A20 + PD-treated cells (Days 15 and 100). (E) As a control for homeostatic driven proliferation (HDP) in a lymphopenic environment, irradiated C57BL/6 mice received PD-treated cells. Heat map analysis was performed for LAG-3 and mapped back to the z-score for each cell. Each group contains 9–15 mice in 3 independent experiments. Cells from multiple animals are included in each plot. (TIF) [file pone.0234778.s005.tif]

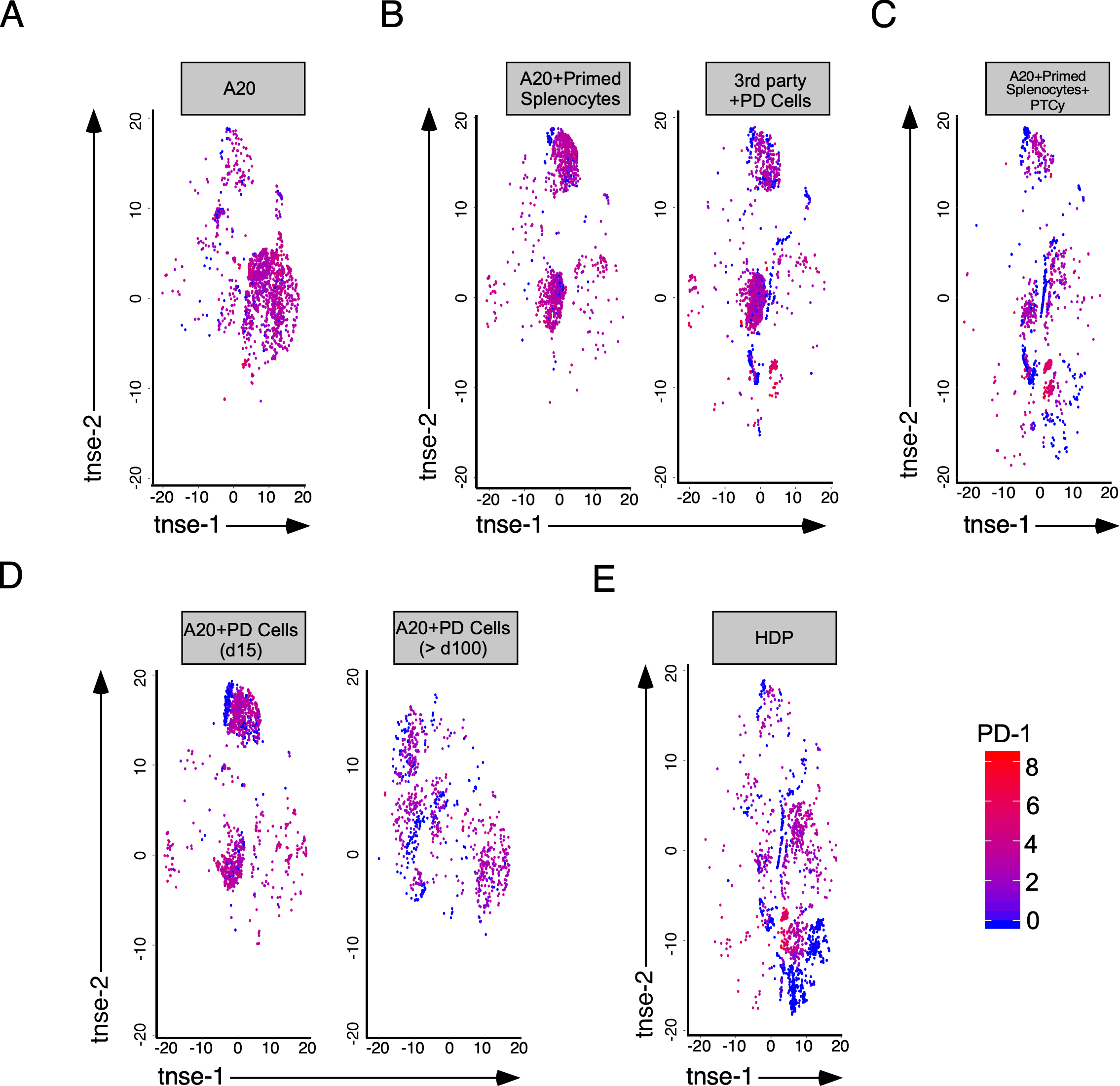

Supplement: S6 Fig — t-SNE transformation was performed on the high-dimensional FACS data obtained from the analysis of isolated PBMCs from recipient mice 15 or 100 days after HCT. The immune signatures are shown for mice that received (A) A20 leukemia alone, (B) A20 + primed splenocytes, or for 3rd- party recipients of PD-treated cells, (C) A20 + primed splenocytes + PTCy, and (D) A20 + PD-treated cells (Days 15 and 100). (E) As a control for homeostatic driven proliferation (HDP) in a lymphopenic environment, irradiated C57BL/6 mice received PD-treated cells. Heat map analysis was performed for PD-1 and mapped back to the z-score for each cell. Each group contains 9–15 mice in 3 independent experiments. Cells from multiple animals are included in each plot. (TIF) [file pone.0234778.s006.tif]
